# Green, Eco-Friendly, Highly Biocompatible and Bioactive Nanocomposite-Based Biopolymers Loaded with ZnO@Fe_3_O_4_ Nanoparticles

**DOI:** 10.3390/polym15173641

**Published:** 2023-09-04

**Authors:** Ayed S. Allogmani, Roushdy M. Mohamed, Mohamed S. Hasanin

**Affiliations:** 1 University of Jeddah, College of Science and Arts at Khulis, Department of Biology, Jeddah, Saudi Arabia; 2 Cellulose and Paper Department, National Research Centre, Dokki, Cairo 12622, Egypt

**Keywords:** nanocomposite, bimetallic, biocompatibility, antimicrobial activity, cytotoxicity activity

## Abstract

Biocompatibility is a major concern for promising multifunctional bioactive materials. Unfortunately, bioactive materials lack biocompatibility in some respects, so active ingredient formulations are urgently needed. Bimetallic nanoparticles have demonstrated drawbacks in stabilized biocompatible formulations. This study examined the preparation of biomaterial-based multifunctional biopolymers via an eco-friendly formulation method using ultrasound. Bimetallic zinc oxide/iron oxide (magnetic form) nanoparticles (ZnO@Fe_3_O_4_NPs) were formulated using casein and starch as capping agents and stabilizers. The formulated nanocomposite was characterized using ultraviolet–visible spectroscopy (UV-vis), Fourier-transform infrared spectroscopy (FTIR), X-ray diffraction (XRD), thermal gravimetric analysis (TGA), scanning electron microscopy (SEM), and high-resolution transmission electron microscopy (HR-TEM). Herein, the formulated nanocomposite was shown to have a thermally stable nanostructure, and the bimetallic ZnO@Fe_3_O_4_ NPs were measured as 85 nm length and 13 nm width. Additionally, the biocompatibility test showed its excellent cytocompatibility with Wi 38 and Vero normal cell lines, with IC_50_ 550 and 650 mg/mL, respectively. Moreover, the antimicrobial activity was noted against six pathogens that are represent to the most common pathogenic microbes, with the time required for killing of bacteria and unicellular fungi being 19 h and 61 h for filamentous fungi with remarket an excellent antioxidant activity.

## 1. Introduction

Microbial infections are defined as the invasion of tissues by pathogens, and the host tissues respond to the infectious agent that sometimes produces toxins that are released into surrounding areas [1,2]. Furthermore, a sickness brought on by an infection is referred to as an infectious disease, which is often known as a transmissible disease or communicable disease [3]. Many different pathogens can cause infections, but the bacteria are the most common causative agents that affect the host, as well as posing a risk of a major secondary infection [4]. Mammalian hosts respond to infections by first going through an innate response, which frequently involves inflammation, and then going through an adaptive response [5]. 

Herein, the treatment of microbial infections was carried out using antibiotics, antivirals, antifungals, antiprotozoals, and anthelmintics. Indeed, more than 9.2 million people died from infectious diseases in 2023, which means that more than 17% of all deaths were caused by infectious diseases, referring to the area of medicine that focuses on infections [6,7]. 

Unfortunately, antimicrobial agents are usually recorded as having dangerous side effects on normal cells, which means the specificity and selectivity of promising agents sometimes cannot be controlled. Therefore, the formulation of biological agents or drugs is a recommended strategy not only to reduce those side effects, but also to improve the role and dosage design [8,9]. 

In this context, a bimetallic nanoparticle could overcome the multiresistance of microbial infections as well as cause a nice synergetic effect between the bimetallic metals that largely amplify the antimicrobial activity [10]. Various methods were used to synthesize nanoparticles that are listed as chemical, physical, and biological techniques [11]. The chemical method is not recommended due to environmental issues. However, the biological and physical methods are recognized as green processes for ZnONPs synthesis, whereas the biological method is still limited in terms of productivity [12]. Moreover, the in situ synthesis of bimetallic nanoparticles using physical methods is noted in the literature as excellent at meeting energy and equipment needs [13].

When employed as an adjuvant therapy to chemotherapy medications, zinc oxide nanoparticles (ZnONPs) can have a variety of therapeutic benefits, including antibacterial, anticancer, immunomodulatory, sunscreen, and antioxidant properties [14]. ZnONPs are extensively used as dermo-compatible and editable compounds, and also it is recommended as antimicrobial and anticancer agent with good cytocompatibility properties [15] and promising photoactivity that supports biological activity mechanism [16]. On the other hand, the use of Fe_3_O_4_ nanoparticles in several novel fields will be highlighted, including energy storage, biosensing, environmental applications for the removal of heavy metals and organic pollutants, moreover it is use in development of magnetic resonance imaging (MRI) contrast agents, as well as antimicrobial and anticancer drug delivery agents with a good biosafety profile [17,18]. Moreover, Fe_3_O_4_ nanoparticles are characterized by unique physicochemical properties, such as those of magnetic and electrical properties [19]. 

Indeed, ZnO@Fe_3_O_4_ NPs exhibit good antimicrobial activity that are relevant to the synergetic effect of the antimicrobial activity of both metals, which are induced by a higher free energy change, leading to the increased generation of the reactive oxygen species (ROS) [20,21]. Undoubtedly, bimetallic nanoparticles are an innovative notion that is acceptable due to their excellent multifunctionality that has been reported in the last few years [22,23,24]. In particular, the biocompatibility process plays an important role in the usability of bimetallic nanoparticles, and the formulation of bimetallic nanoparticles are usually recommended to enhance their stability and usability, while decreasing or eliminating the side effects as well [25]. 

Biopolymers are usually editable, compatible, and non-toxic because they are produced mainly by biological systems [26,27]. One of the biopolymers that can be separated from both human and animal milk is casein [28]. Evidently, this renders casein essentially non-toxic and compatible. In addition, casein is chemically characterized as a phosphoprotein that is chemically active and adaptable [29]. Additionally, one of the most significant edible biopolymers used globally is starch. The two forms of alpha-glucan polysaccharide that make up the majority (98–99%) of starch are amylose (-(1-4)) and amylopectin (-(1-6)) in granules made of glucose units [30]. Here, the bioactive nanocomposite that is decorated with nanometals could exhibit strong antimicrobial, anticancer, and antioxidant activities, all while maintaining biocompatibility [31,32,33,34,35].

Evidently, the chief disadvantages of prepared nanoparticles are their synthesis approach, their instability, and the cytotoxicity they cause due to their ability to be easily released. Furthermore, biocompatibility has a limited impact on whether the formulated materials will be accepted or rejected for biomedical applications, as incompatible materials can damage cells and tissues, resulting in destruction [36,37,38]. Therefore, the present work offers an eco-friendly approach to fabricate a completely biocompatible, antimicrobial, and antioxidant nanocomposite based on biopolymers like casein and starch encapsulated with ZnO@Fe_3_O_4_ NPs, which are synthesized in situ, with the biopolymers acting as capping and stabilizing agents. The prepared nanocomposite was characterized using advanced techniques to affirm the structure, including physiochemical and topographical analyses. Additionally, the biological profile will include antimicrobial, release, cytotoxicity, and antioxidant activities.

## 2. Materials and Methods

### 2.1. Materials

Soluble casein and starch were purchased from Sigma-Aldrich Co. (Darmstadt, Germany). Zinc acetate pentahydrate and ferric chloride were purchased from Loba Chem. Co. (Maharashtra, India). All microbial tissue culture media and reagents were purchased from Loba Chem. Co. (India) and were used as received.

### 2.2. Methodology

The formulation of ZNO@Fe_3_O_4_ NPs was carried out in situ during the preparation of nanocomposite-based biopolymers, namely, casein and starch, according to previous studies with minor modifications [39,40]. In detail, one gram of soluble casein was dissolved in 100 mL 1% acetic acid solution. Also, one gram of starch was dissolved in 100 mL of deionized water. Both solutions were individually and vigorously stirred at 1500 rpm at 70 °C for 2 h and mixed while stirring at 1500 rpm for 2 h till the solution became milky brown in color, that indicating the end point of this step. Afterward, the above-prepared solutions were ultrasonicated in an ultrasonic water bath at 70 °C for 2 h. In addition, zinc acetate and ferric chloride solutions were prepared separately with a concentration of 1% (*w*/*v*) in deionized water. The above three preprepared solutions were mixed (total volume 300 mL), whereas the metal salt solutions were added dropwise to avoid aggregation under vigorous stirring at 1500 rpm at 95 °C till the volume decreased to 200 mL. Finally, the collected solution was concentrated again to 100 mL, and then sonicated with an ultrasonic probe for 10 min. The preprepared nanocomposite was washed several times with 70% alcohol and centrifuged at 1000 rpm at least three times before lyophilization and preservation in a refrigerator for further investigations.

### 2.3. Characterizations

The characterizations of the prepared nanocomposite were carried out via physiochemical and morphological analyses and compared with those of neat materials. Physiochemical analysis involved an Ultraviolet–visible (UV-Visible) spectrophotometer (Jasco, V-630, Tokyo, Japan) in the range of 200–1000 nm, Fourier-transform infrared spectroscopy (FTIR) using an Impact-400 FT-IR spectrometer (Nicolet Analytical Instruments, 5225-1, Madison, WI, USA) in the range of 400–4000 cm^−1^, and X-ray diffraction (XRD) at a 2θ (Bragg angle) of 5–80° using a Bruker D8 Advance X-ray diffractometer (Karlsruhe, Germany). Additionally, in this morphological study, transmission electron microscopy (TEM) was carried out using a high-resolution electron microscope (HRTEM, JEOL 2010, Tokyo, Japan) operating at 300 kV and selected area electron diffraction (SAED). Scanning electron microscope (SEM) images were taken using a scan electron microscope attached with EDX, Model Quanta 250 FEG (Field Emission Gun) attached to an EDX Unit (Energy Dispersive X-ray Analyses), and thermal stability analysis was carried out in a nitrogen atmosphere with a heating rate of 10 °C/min using an SDT Q600 thermal analyzer, TA Instruments, New Castle, DE, USA.

### 2.4. Biological Profile

The biological profile study was included a cytotoxicity assay, antimicrobial activity and antioxidant activity. The cytocompatibility test of the nanocomposite was carried out using the MTT protocol [41] with minor modifications against the human normal cell line, Wi 38, and Vero fibroblast normal cells were collected from the American type culture collection (ATCC). The cell quantity and the percentage of viable cells were calculated according to the following equation,

$$Viability\;\%=\frac{Test\;OD}{Control\;OD}\times 100$$

Additionally, the antimicrobial activity study was carried out using the turbidimetric assay, as carried out in our previous work [42], with an initial concentration of 100 µg/mL nanocomposite. The microbes used in this study were Gram-positive (*Bacillus subtilis* ATCC 6051 and *Staphylococcus aureus* ATCC 25923), Gram-negative (*Escherichia coli* ATCC 25922 and *Pseudomonas aeruginosa* ATCC 27853), unicellular fungi (*Candida albicans* ATCC 90028), and filamentous fungi (*Aspergillus niger* RCMB 02724). The time required for the killing assay was calculated according to previous work, with minor modifications [43], as 24 h for bacteria and unicellular fungi and 72 h for filamentous fungi. 

According to [44,45], the DPPH method was used for the antioxidant activity assessment, which was carried out according to the radical scavenging ability. To assess the antioxidant activity behavior of the extracted SNPs, the tested samples were generated in various concentrations with a range of 1–12%.

### 2.5. Statistical Analysis

The means of three replicates and standard errors were calculated for all obtained results, and the data were subjected to analysis of variance means using Minitab 21.2 software.

## 3. Results and Discussion

### 3.1. Formulation of Nanocomposite

The formulation of the nanocomposite was carried out based on casein and starch biopolymers used as capping and stabilizing agents for the green synthesis of ZNO@Fe_3_O_4_ NPs. The formulated nanocomposite was formulated in one step via a green methods, in which the reducing particle size and formulation was carried out.

### 3.2. Characterization of the Prepared Nanocomposite

**Figure 1:**
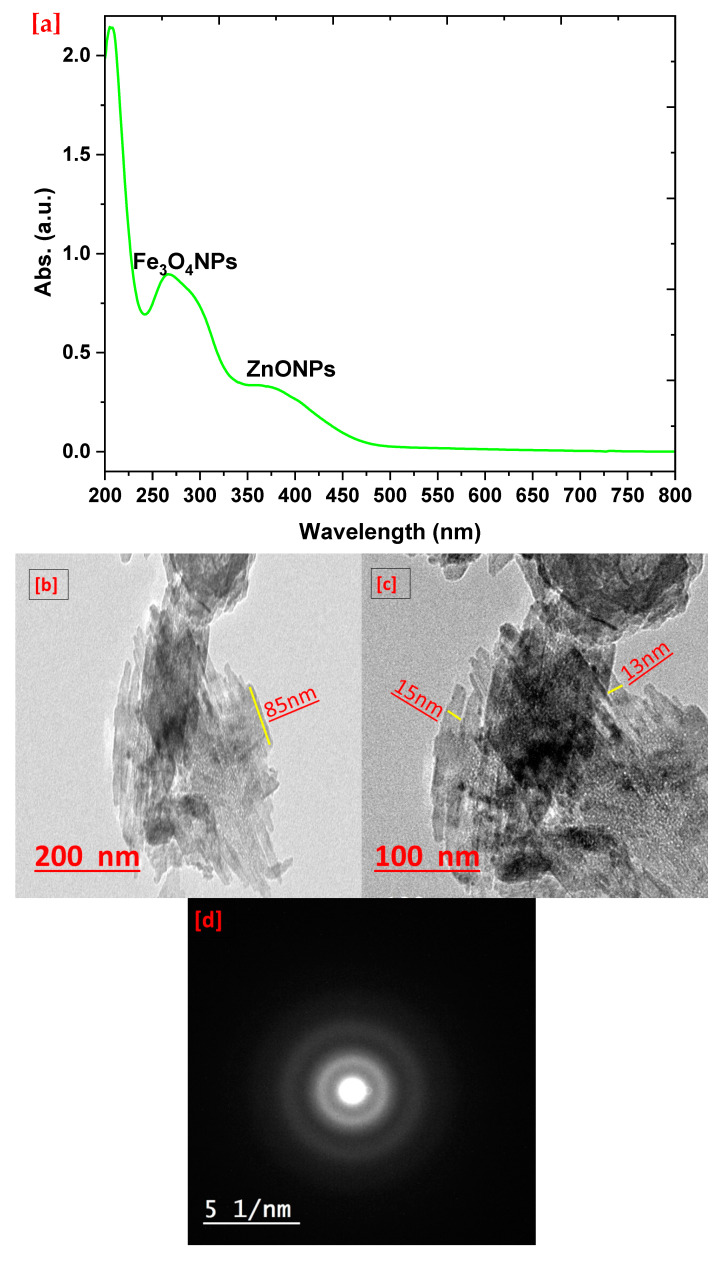
UV-vis of prepared nanocomposite (**a**) and HR-TEM images at low (**b**) and high (**c**) magnifications as well as the SEAD pattern (**d**).

The physicochemical analysis and topographical study were used to evaluate the properties of the nanocomposite, which is sometimes required to compare its neat materials. UV-vis spectroscopy was used to illustrate the performance of the nanoparticles via surface plasmon resonance adsorption peaks that are specific for each nanoparticle, especially metals and metal oxides. Figure 1a illustrates the UV-vis spectrum of the nanocomposite, where two bands were observed at 263 and 374 nm due to Fe_3_O_4_ NPs and ZnO NPs, respectively [46,47]. Obviously, the bands were presented broadly, which could be due to two reasons: the interaction between bimetallic structure and the entrance of bimetallic particles into the biopolymers helix, that affect the surface plasmon resonance. The TEM study reflected the size and arrangement of the nanoparticles, as shown in Figure 1b,c. The nanocomposite typically appeared as nanostructure that consisted of matrix containing bimetallic nanoparticles. Obviously, the matrix was assigned as a nanostructure that connected the bimetallic nanoparticles that had rod shapes arranged together as a strap. Moreover, these nanorods could be referred to as ZnONPs and Fe_3_O_4_NPs [48,49]. However, in regard to binding both metal oxide nanoparticles, the texture of bimetallic particles was recorded as a rough surface containing many scars that could be due to Fe_3_O_4_NPs’ texture, and this observation agrees well with [50]. In this context, the SEAD pattern was observed as a polycrystalline behavior for the nanocomposite, with four rings arranged inside each other that could be as a result for interaction between the biopolymers and bimetallic nanoparticles [51]. In summary, the HR-TEM images emphasize the nanostructure of the nanocomposite, which agrees well with the UV-vis analysis conclusion. In this context, the bimetallic particles were recorded with the following dimensions: 85 nm long and 13 nm wide.

**Figure 2:**
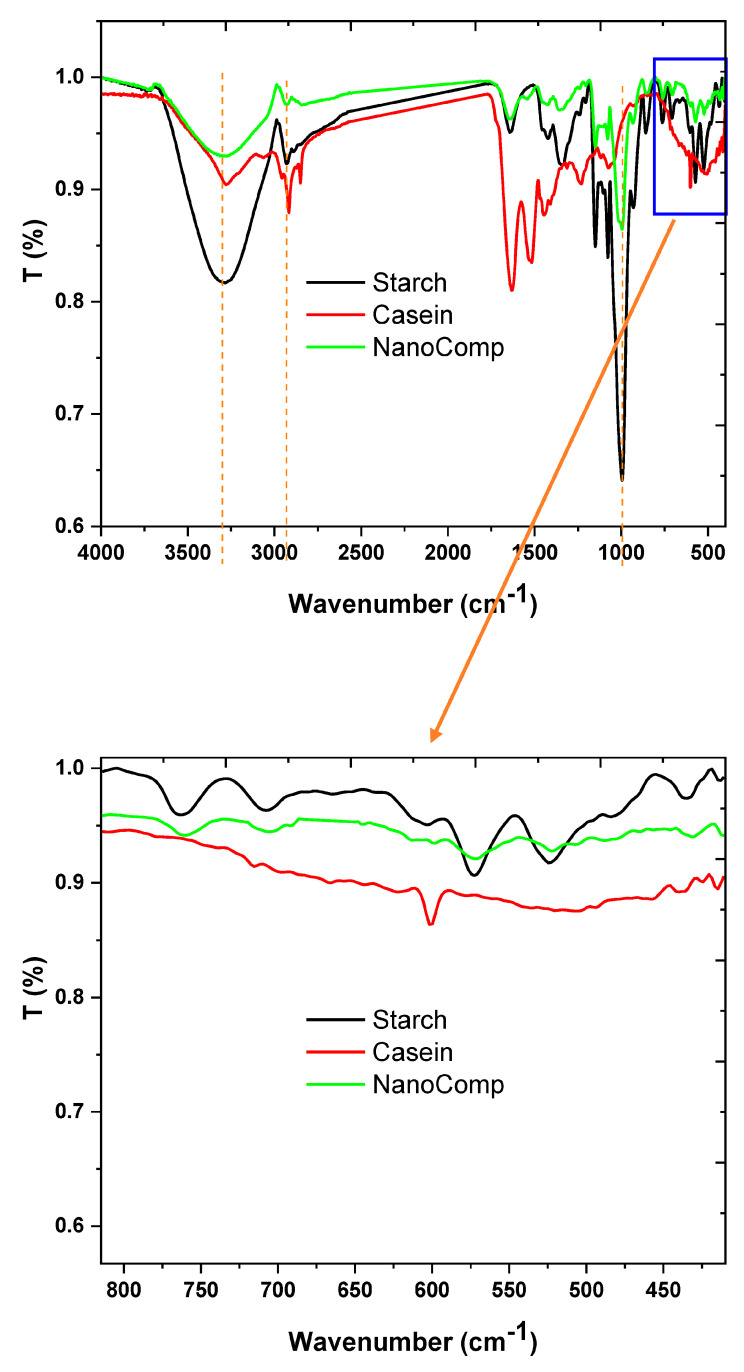
FTIR of nanocomposite and its neat materials.

FTIR spectroscopy was used to assign the function group changes and reactivity, as presented in Figure 2. The starch spectrum presented bands at 3290, 2925, 1635, 1420, 1340, 1008, 870, and 755 cm^−1^, which are corresponding to the stretching vibrations of hydroxyl groups, CH_2_ stretching, H-O-H bending, CH_2_ bending, twisting of α-1,4 glycosidic linkage (C-O-C), C(1)-H deformation, CH_2_ deformation, and C-C stretching, respectively [30,52,53]. Furthermore, the casein spectrum showed characteristic bands at 3281 and 3092 cm^−1^, corresponding to the overlapping of amide A with hydroxyl group vibrations and amide B, respectively [54,55]. Additionally, the bands at 2956, 2915, and 2850 cm^−1^ were assigned to different C-H stretching and bending modes. The bands at 1635 and 1520 cm^−1^ are referred to as amide I, and a band at 1065 cm^−1^ for C-O-C of carboxylate compounds was recorded at 1446 cm^−1^ [54,56].

On the other hand, the nanocomposite spectrum reflected more significant changes in comparison with those of its neat materials, not only in the bands’ positions, but also in the bands’ shape and intensity, which affirms the combination and excellent compatibility between the nanocomposite components at a molecular level. The band at 3290 cm^−1^ appeared as a result of the interaction between starch (-OH) and casein (-NH), which was broader than both, and the band of CH stretching was assigned to 2925 cm^−1^ as a small band. Moreover, the amide I band was shifted to a higher frequency, which may be due to the interaction between starch (-OH) and casein (-NH) that increases the negative charge around the amide group and increases the frequency as well. Additionally, the carbohydrate band was assigned at approximately at the same position, but with a low intensity. Moreover, the bimetallic nanoparticles were noted as small bands at 428 and 407 cm^−1^, which referred to Zn-O [57], and Fe-O bands were recorded at 641 and 580 cm^−1^ [58], and the overlapped areas between both nanoparticles were noted as small bands at 690, 508, and 446 cm^−1^ [59]. Clearly, the FTIR study affirmed that the nanocomposite at the molecular level was prepared with new features that were different than those of its parent component.

**Figure 3:**
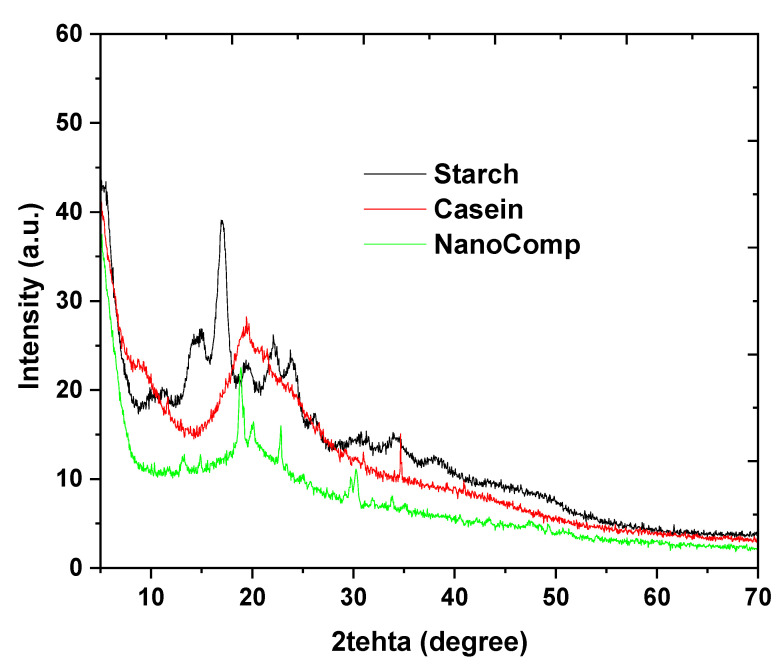
XRD patterns of nanocomposite and its neat materials.

The crystallographic analysis of the nanocomposite and its parent materials is presented in Figure 3. XRD was used to perform crystallography for the materials that were significantly changed after being doped with nanometal and nanometal oxides. The starch pattern referred to characteristic peaks at 2θ = 11. 15, 17, 19, 22, and 24° that related to native starch crystallography values [52,60,61]. The casein pattern was shown peaks at 2θ = 9 and 21°, where this peak and pattern behavior was typical with neat casein [62,63,64]. In addition, the nanocomposite pattern presented peaks that represented to the biopolymers interactions at 2θ = 13, 15, 19, 20, 23, and 25°, where these peaks noted in pattern of the pure starch and casein, whereas other peaks were noted at 2θ = 30, 32, 34, 36, 43, 47, 49, and 55° that were referred to the overlapping of ZnONPs and Fe_3_O_4_ [65,66,67,68]. Indeed, crystallographic analysis emphasized that the bimetallic nanoparticles well matched with the card number (JCPDS FILE No., 06–362) for ZnONPs [69] and (JCPDS98-0625) for Fe_2_O_3_ [46], with a minor difference that could be due to interactions between bimetallic particles as well as the formulation of the nanocomposite’s new network, which may affect the molecular structure of the particles. These assumptions agree well with the UV-vis and FTIR studies’ conclusions.

**Figure 4:**
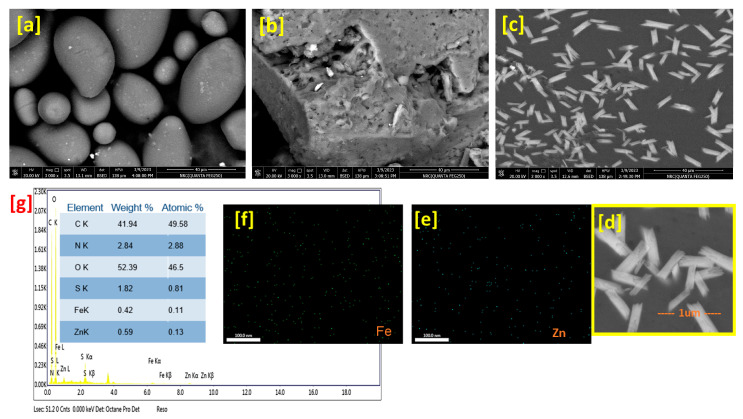
SEM images of neat starch (**a**), casein (**b**) and nanocomposite at low (**c**) and high (**d**) magnifications as well as mapping of Zn atom (**e**), Fe atom (**f**), and EDX chart (**g**).

Figure 4 presents SEM images of the nanocomposite and its neat components as well as the mapping and EDX chart of the nanocomposite. The SEM study reflects the surface behaviors, the EDX data present the elemental analysis of the samples, and the map affirms the elemental distribution. Figure 4a shows the native starch image that performed atypical morphology of pure starch with a unique spherical shape. Additionally, the casein surface (Figure 4b) appeared as a bulky structure, which is related to a conventional casein surface, with some pores that give the surface morphology a rough texture. In this context, the nanocomposite image at low magnification (Figure 4a) was observed as a smooth surface decorated with metal-like rods. However, the highly magnified image (Figure 4d) appeared a small rods collected together as a straps in a matrix, over which these rods could be collected or attached, contained holes. Herein, these observations affirm the TEM study conclusions as well. Nevertheless, the EDX chart (Figure 4g) shows the elemental analysis of the nanocomposite, which contains carbon, oxygen, nitrogen, sulfur, iron, and zinc. Furthermore, the mapping images of Zn and Fe in Figure 4e,f, respectively, were shown that the bimetallic particles offer an excellent distribution over the nanocomposite matrix. 

**Figure 5:**
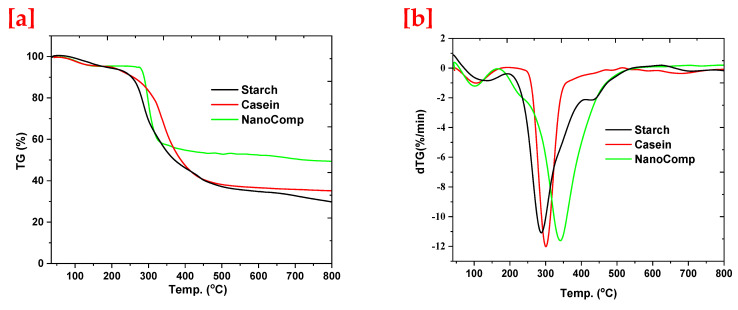
Thermal analysis of neat starch, casein, and nanocomposite TGA (**a**) and DTGA (**b**).

The thermal behavior of the nanocomposite was studied using TGA and DTGA, and then was compared to its parent components, as presented in Figure 5. It is well known that thermal analysis studies not only refer to thermal stability, but also affirms the structure’s stability. The TGA charts presented in Figure 5a, that presented a thermal stability of nanocomposite in comparison with its parent materials, as well as showed remaining weight refers to metal oxide nanoparticles. Otherwise, the decomposition of the nanocomposite was delayed in comparison with that of starch and casein. On the other hand, the DTGA data (Figure 5b) illustrate the main decomposition stage and the recorded peaks for starch, casein, and nanocomposite at 289, 303, and 345 °C, respectively. In this context, the stage of decomposition of pure starch started at 203 °C and ended at 416 °C, and for casein started at 238 °C and ended at 358 °C, whereas the nanocomposite recorded a starting decomposition temperature at 226 °C, that dropped, recovered, and then restarted at 246 °C and ended at 529 °C. These observations, especially for the nanocomposite, were due to the reconfiguration of the molecular structure after it was imbedded with nanoparticles, so that, at first, small peaks adsorbed the temperature according to thermal conductivity and configurated with the polymer matrix, which affected the thermal stability positively [70,71]. 

### 3.3. Biological Profile

The biological profile of the prepared nanocomposite was tested against microbial pathogens, including bacteria (Gram positive and negative) and fungi (unicellular and filamentous). Additionally, the biocompatibility property was tested using the Wi 38 human normal cell line and Vero fibroblast cell lines. Also, an antioxidant activity assay using DPPH was carried out to evaluate the complementary biological profile of the formulated nanocomposite. 

#### 3.3.1. Cytocompatibility Test

**Figure 6:**
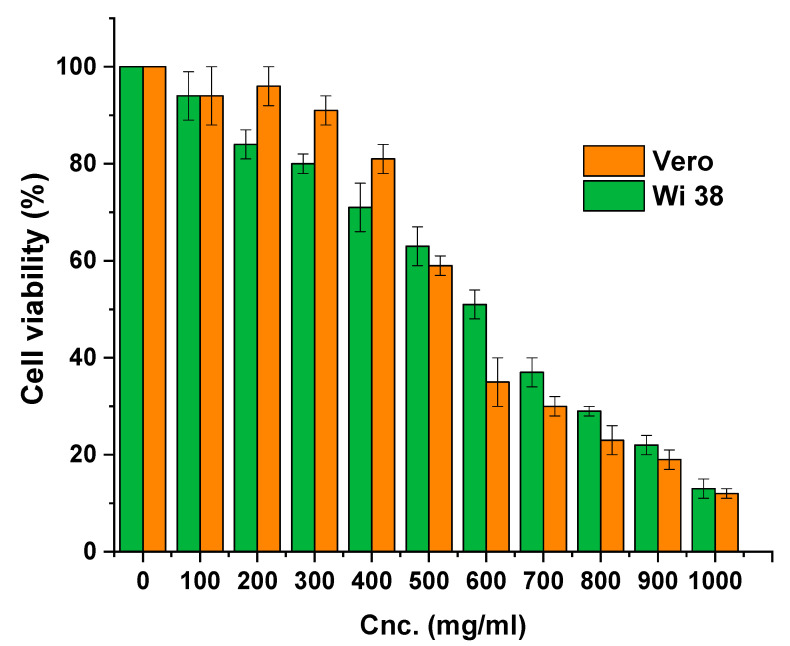
The cytocompatibility test was performed using the Wi 38 human normal cell line and Vero fibroblast cell lines against formulated nanocomposite.

The cytocompatibility test was performed using the Wi 38 human normal cell line and Vero fibroblast cell lines, as presented in Figure 6. The produced nanocomposite demonstrated cytocompatibility up to 550 and 650 mg/mL for the Wi 38 and Vero cells, respectively, after exposure to concentrations of 100–1000 mg/mL. Moreover, the IC_50_ was 480 µg/mL for Wi 38, and that for the Vero cells were recorded as 450 mg/mL, which is regarded as having great cell compatibility. Furthermore, at high concentrations (greater than 500 mg/mL), the cell was healthy and did not exhibit any signs of significant deformation, shrinkage, or swelling. This indicates that the high concentration of the nanocomposite did not affect the cells directly, but rather the composition of the culture medium as a result of the adsorption of medium nutrients that affected a growth number of cells [72,73]. Overall, the obtained results affirmed that the prepared nanocomposite has high cytocompatibility, which means low cytotoxicity.

#### 3.3.2. Antimicrobial Activity Study

**Figure 7:**
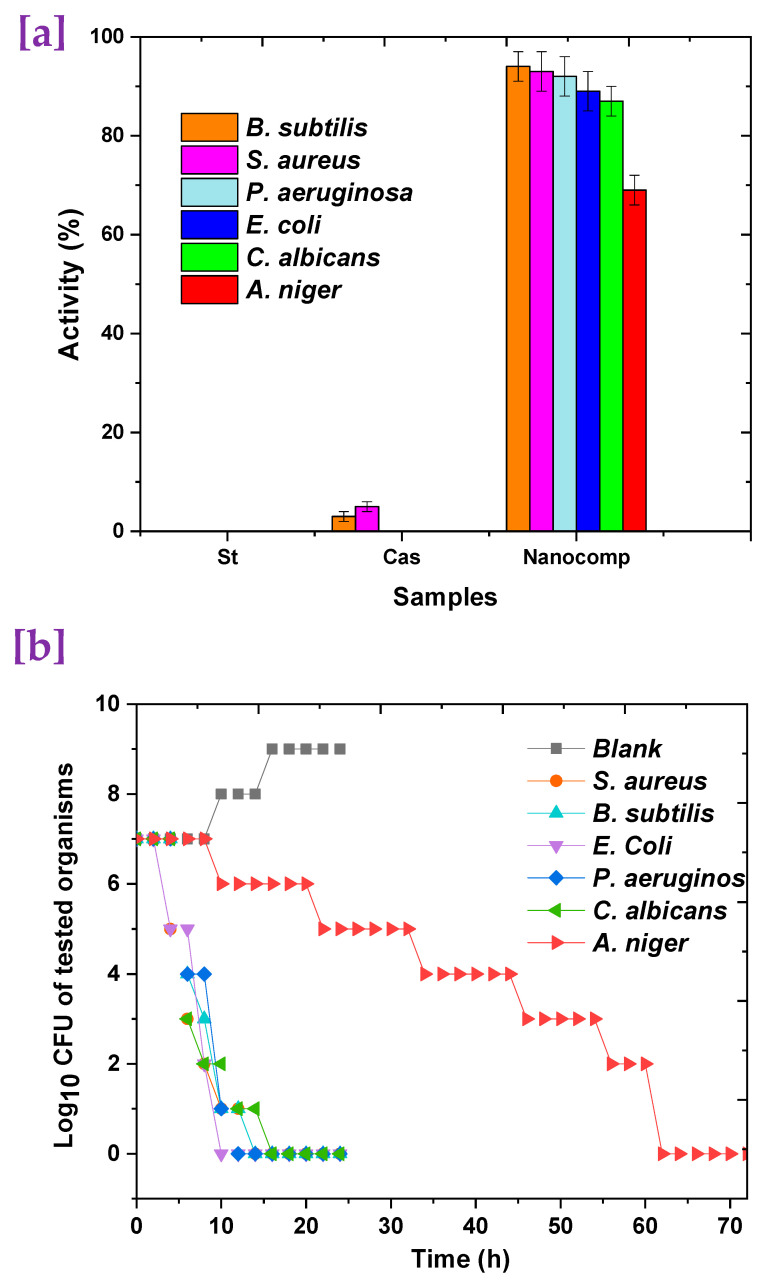
Antimicrobial study of formulated nanocomposite: (**a**) antimicrobial activity and (**b**) the time required for killing.

Six pathogenic microorganisms representing Gram-positive, Gram-negative, unicellular, and filamentous fungi were used in an antimicrobial investigation, as illustrated in Figure 7. Obviously, the formulated nanocomposite presented excellent antimicrobial activity against all the microbial populations recorded, such as 54% for filamentous fungi (Figure 7a). According to the mode of action, antimicrobial activity was gained from synergetic ZnO@Fe_3_O_4_, which has wonderful antibacterial activity that is pertinent to the synergetic impact of the antimicrobial activity of both metals, which is triggered by a larger free-energy change, leading to the increased formation of reactive oxygen species (ROS) [20,21,74]. Additionally, casein plays a role in antimicrobial activity via binding with the growth regulators of microbes as electrolytes and deactivating cell progression [75].

Moreover, the formulated nanocomposite was investigated to estimate the time required for killing and eradicating various microbial populations, as shown in Figure 7b. The killing rate of the bacterial strains was presented a slow rate during the first 5 h, thereafter the rate was faster, and the elimination of all Gram-negative bacterial cells after 16 h. Additionally, Gram-negative bacteria had a high death rate, but this occurred over a longer period than that of the positive ones. Furthermore, all bacterial strains had finished killing after 18 h, and this rate is regarded as high. In addition, the killing rate of unicellular fungi was detected as slower than that of bacteria, where the time needed to kill was found to be 19 h. However, the filamentous fungi showed the lowest rate of killing during the first 8 h, but the pace accelerated after 12 h, reaching a plateau. Since the rate of growth coincides with the death rate, the killing rate of filamentous fungi observed at 61 h was slower than that of unicellular fungi. Overall, it was discovered that the prepared nanocomposite had a promising death rate and efficacy against all microbiological populations.

#### 3.3.3. Antioxidant

**Figure 8:**
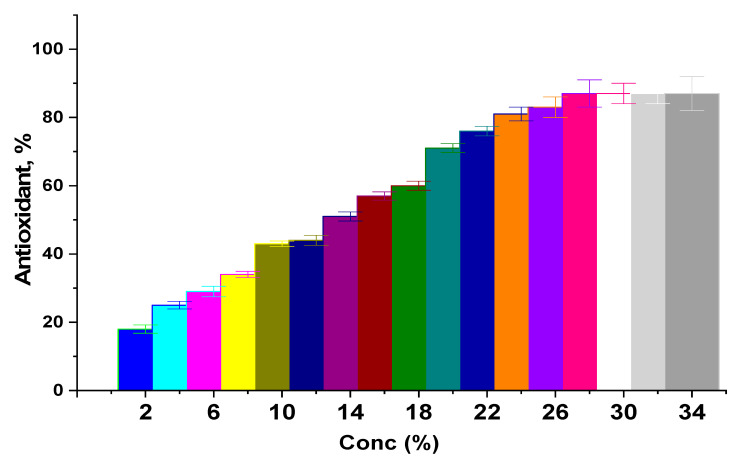
Antioxidant activity of formulated nanocomposite.

The formulated nanocomposite underwent an antioxidant test to determine how well it functioned as an antioxidant substance. The antioxidant properties are shown in Figure 8 with different concentrations of the nanocomposite: 0–34% (*wt*/*v*). Consequently, the structure and the activity of the nanocomposite affected the DPPH reagent; the activity was reached 85% at concentrations of 28%. The obtained results highlight the antioxidant activity of the nanocomposite, which increases concurrently with the concentration of nanocomposite. Indeed, the antioxidant properties were gained from starch [30], casein [76], and bimetallic nanoparticles [77,78] as well. Indeed, nanoparticles play a potential role in increasing the capacity of DPPH scavenging. Generally, the radicals generated during oxidation are a great way to combine the huge surface area of nanoparticles and reduce the oxidative activity of the free radicals [79]. In addition, starch has a role in inhibitory activity, thereby preventing the biochemical pathways that trigger the production of free radicals inside mitochondria [80]. Additionally, casein has a mechanism of antioxidant action, which forms a complex structure with free radicals and regulates their equilibrium to prevent oxidative reactions [81].

These outcomes not only highlight the antioxidant properties of the nanocomposite, but also support the effectiveness of the extraction technique, which preserve the distinctive characteristics of the generated nanocomposite.

## 4. Conclusions

The formulation of bimetallic nanoparticles into biopolymers was successfully achieved through a nanocomposite, according to physicochemical and topographical studies. Additionally, the formulated nanocomposite presented a nice cytocompatibility, with excellent IC50s as 550 and 650 mg/mL for Wi 38 and Vero cells, respectively, as well as a broad spectrum of antibacterial activity against Gram-positive and Gram-negative bacteria. Moreover, antifungal activity was assigned against unicellular and filamentous fungi, with a fast rate of killing. Antioxidant activity was observed at a concentration of 28% at about 85%. The formulated nanocomposite could be recommended to use as a multifunctional biologically active material for different biomedical applications.

## Data Availability

The datasets generated during and/or analyzed during the current study are available from the corresponding author upon reasonable request.
